# Exploring experiences of cancer care in Wales: a thematic analysis of free-text responses to the 2013 Wales Cancer Patient Experience Survey (WCPES)

**DOI:** 10.1136/bmjopen-2016-011830

**Published:** 2016-09-01

**Authors:** Michael Bracher, Dame Jessica Corner, Richard Wagland

**Affiliations:** Faculty of Health Sciences, University of Southampton, Highfield, Southampton, UK

**Keywords:** cancer, patient experience, QUALITATIVE RESEARCH, free text, wales, outcomes

## Abstract

**Objectives:**

To provide the first systematic analysis of a national (Wales) sample of free-text comments from patients with cancer, to determine emerging themes and insights regarding experiences of cancer care in Wales.

**Design:**

Thematic analysis of free-text data from a population-based survey.

**Setting and participants:**

Adult patients with a confirmed cancer diagnosis treated within a 3-month period during 2012 in the 7 health boards and 1 trust providing cancer care in Wales.

**Main outcome measures:**

Free-text categorised by theme, coded as positive or negative, with ratios. Overarching themes are identified incorporating comment categories.

**Methods:**

4672 respondents (of n=7352 survey respondents) provided free-text comments. Data were coded using a multistage approach: (1) coding of comments into general categories (eg, nursing, surgery, etc), (2) coding of subcategories within main categories (eg, nursing care, nursing communication, etc), (3) cross-sectional analysis to identify themes cutting across categories, (4) mapping of categories/subcategories to corresponding closed questions in the Wales Cancer Patient Experience Survey (WCPES) data for comparison.

**Results:**

Most free-text respondents (82%, n 3818) provided positive comments about their cancer care, with 49% (n=2313) giving a negative comment (ratio 0.6:1, negative-to-positive). 3172 respondents (67.9% of free-text respondents) provided a comment mapping to 1 of 4 overarching themes: communication (n=1673, 35.8% free-text respondents, a ratio of 1.0:1); waiting during the treatment and/or post-treatment phase (n=923, 19.8%, ratio 1.5:1); staffing and resource levels (n=671, 14.4% ratio 5.3:1); speed and quality of diagnostic care (n=374, 8.0%, ratio 1.5:1). Within these areas, constituent subthemes are discussed.

**Conclusions:**

This study presents specific areas of concern for patients with cancer, and reveals a number of themes present across the cancer journey. While the majority of comments were positive, analysis reveals concerns shared by significant numbers of respondents. Timely communication can help to manage these anxieties, even where delays or difficulties in treatment may be encountered.

**Strengths and limitations of this study**

- Provides further detail on closed measures in population-based survey.
- Indicates area of concern not addressed by closed measures.
- Volume of comments and ratios of negative-to-positive comments in specific areas indicate areas of particular concern.

## Introduction

The global burden of cancer disease is growing worldwide.^1^ Increasing numbers of people in the UK are affected by cancer diagnosis and treatment, with lifetime risk being projected at one in two for those born from the early 1960s onward.^2^ However, research has indicated that survival rates for all cancers combined has increased substantially since 1971, and that more people are living longer with, and beyond, their cancer.^3^ Patient experiences of cancer treatment and support, through diagnostic, treatment and post-treatment phases, are therefore areas of significant and growing public concern.

There is increasing recognition in Europe and North America that the quality and effectiveness of services are best evaluated from the patient's perspective.^4^ Patient-reported outcome measures and experience measures are commonly used to explore patients' views on their care and treatment,^5^ ^6^ and frequently include open-ended questions. Open questions can enhance understanding of responses to closed questions by providing greater detail on experiences, and allowing respondents to offer information that may not be elicited through closed measures. However, these data often remain unexplored, due to the time and resource-heavy process of analysing large free-text sets.^7^

In the UK, the National Health Service (NHS) Cancer Reform Strategy and Outcomes Strategy for Cancer documents^8^ highlight the important role of patient experiences in measuring and improving clinical quality, and national surveys have been undertaken to determine the quality of experience of patients with cancer and survivors.^9–11^ In England, the Cancer Patient Experience Survey (CPES) has been conducted annually since 2010, and continues to provide useful insights into patient experiences of cancer treatment and care.^12^ Data from responses to closed questions in this survey have been used in previous work by Bone *et al*^13^ to explore variations in overall satisfaction with care by sociodemographic, patient, clinical and trust-related factors. Elsewhere, analysis of free-text responses to the CPES from patients identified with London trusts has been undertaken by Wiseman *et al*.^14^ In 2012, the Cancer Delivery Plan for NHS Wales has recognised the important of patient experience and established a commitment to produce a national survey.^15^ The first Wales Cancer Patient Experience Survey (WCPES) was conducted in 2013 through a partnership between the Welsh Government and Macmillan Cancer Support, and was administered by Quality Health. In common with the England CPES, closed questions in the Wales survey related to a number of topic areas, for example, seeing your general practitioner (GP); diagnostic tests; clinical nurse specialist; support for people with cancer.^16^ The majority of respondents related positive experiences of their care; however, there also exist groups of patients who report less positive experience in a variety of areas.

The present study was commissioned by Macmillan Cancer Support to analyse the content of the free-text responses, provide more information on specific experiences of care and treatment, identify any areas that had not been covered by quantitative measures, and thereby facilitate mixed-methods descriptive analysis of the data.

## Methods

### Study design

This investigation involves analysis of secondary data from a population-based postal survey undertaken in Wales in 2013 of all individuals aged ≥16 years with a primary diagnosis of cancer,^i^ who were admitted to an NHS hospital as an inpatient or as a day case patient, and were discharged from hospital between 1 September 2012 and 30 March 2013 (n=10 945).^12^ Survey results were published in January 2014^16^ with a response rate of 69% (n=7352 patients). Results from the closed questions demonstrate a positive experience of cancer care in Wales, with 89% of patients rating their care as excellent (58%) or very good (31%).

### Cohort identification

The seven health boards and one trust treating adult patients with cancer in Wales were included. Patients were identified from data provided by health boards/trusts, selected from local patient administration systems.^16^

### Questionnaire and design content

Questionnaires included questions on sociodemographics, quality of treatment and care, disease status and long-term conditions.^16^ Three free-text comment boxes were placed at the end of the questionnaire, following the closed questions: ‘Was there anything particularly good about your NHS cancer care?’; ‘Was there anything that could have been improved?’; ‘Any other comments?’ (these questions are identical to those used in the 2013 CPES for England).

### Survey process

The survey was distributed by post, with two reminders sent out to non-responders only.^16^ Covering letters were sent out on health board/trust headed paper and signed by a member of the health board/trust staff.^16^ Survey and covering letters were sent out in English and Welsh language versions. An enclosed language leaflet offered translation services and a prepaid return envelope was included so patients could respond without financial cost.^16^ In total, 4576 free-text respondents used English language (63.6% of total English language respondents to the full survey^ii^), while 96 (59.3% of Welsh language respondents^iii^ to the full survey) provided free-text responses in Welsh language, which were translated into English for analysis.

### Ethics and governance

The survey was performed as a service evaluation. Survey respondents had access to a telephone support line to discuss issues raised by the survey.

## Analysis

**Figure 1:**
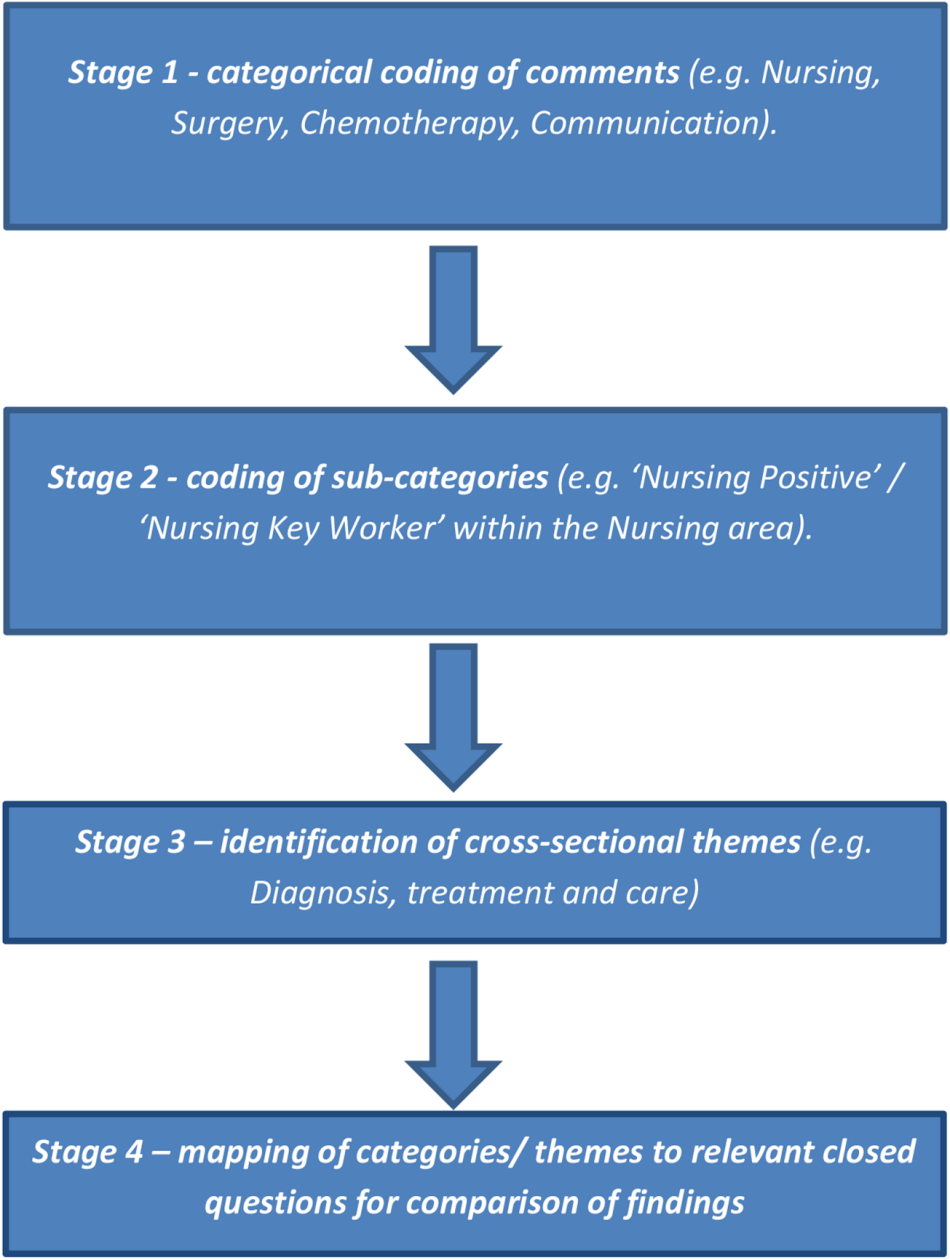
Process of multistage coding.

Data were subjected to a thematic analysis, informed by multistage coding (see figure 1) of free-text data.^17^ The coding taxonomy was developed inductively from the data using the NVivo Qualitative data analysis software package.

*Stage 1*: Stage 1 involved analysis of semantic content of the entire free-text data set (ie, whether comments contained references to nursing care, surgery, chemotherapy, etc) and whether comments^iv^ were of a positive or negative nature. A coding taxonomy was produced for sorting qualitative data into categories of patient experience, developed by one researcher (MB) in collaboration with two further researchers (RW and DJC; see online supplementary appendix table S1). Once the main taxonomy had been established (ie, it accommodated the majority of comments without need for additional categories), a sample of 200 randomly selected comments was double-coded by two researchers (MB and RW). Coding agreement between the researchers was 80% (Cohen's κ), and conflicts were resolved through discussion.

10.1136/bmjopen-2016-011830.supp1 Supplementary tables

*Stage 2*: Individual categories were subjected to a second stage of more detailed sorting into subcategories. For example, at stage 1, comments relating to nursing care were coded to the categories ‘nursing’ and then ‘nursing positive’ or ‘nursing negative’. At stage 2, comments within these categories were sorted further according to what specifically was ‘positive’ or ‘negative’ about the care (eg, patient perceptions of information provided by nurses or the manner in which they were dealt with by staff).^v^

*Stage 3*: Categories/subcategories were subjected to cross-sectional analysis to highlight common themes present across different categories (eg, communication; see online supplementary appendix table S2).

*Stage 4*: Comparisons between results of closed question and free-text responses were conducted where there was appropriate correspondence.

## Findings

In total, 4672 patients provided free-text comments in the survey, representing 64% of those who returned questionnaires. Self-reported demographic data on age, sex, long-standing condition, employment status, ethnicity and sexual orientation were collected (data on tumour type was provided through local patient administration systems). The profile of participants who provided free-text comments was representative of all WCPES respondents (n=7352; see online supplementary appendix table S3). Most free-text respondents (82%, n=3818) provided a positive comment about their cancer care, with 49% (n=2313) providing a negative comment, giving an overall positive ratio of 0.61:1 (see online supplementary appendix table S1).

Stage 3 analysis identified four major overarching themes, incorporating the majority of stage 1 and 2 text categories: *communication; waiting; staffing and resource levels; speed and quality of diagnostic services*. In total, 3172 respondents (63.9% of total free-text respondents) provided a comment relating to one of the four themes identified, of which 1948 (41.7% of total free-text respondents) were negative, and 1276 (27.3% of total free-text respondents) were positive (overall negative ratio of 1.53:1; see online supplementary appendix table S2).

### Communication

The largest single theme was *communication* (1673 respondents) with a balanced ratio of 1.01:1, representing 35.8% of free-text respondents and 22.8% of survey respondents. Comments relating to communication fell into two subthemes; *communication between patients and staff*; and *communication between staff and/or institutions* (ie, sharing information).

#### Communication between patients and staff

A third (31.5%) of free-text respondents (n=1472) provided a comment relating to communication with healthcare staff, of which 854 were negative and 846 positive (a balanced ratio of 1.01:1; see online supplementary appendix table S2). Responses in this subtheme were of two broad types: those commenting on the *quality and/or availability of information provided by staff*; and those referring to the *manner in which patients perceived that they had been dealt with by staff*.

##### Quality and/or availability of information provided by staff

Comments on information provision cut across a wide range of treatment types and staff areas/specialties, with the majority of negative responses related primarily to the availability of adequate information on treatment/care (269 respondents, 5.7% of free-text respondents).

Sometimes it feels like you have to tease information out of doctors—it doesn’t seem to be given readily, you just have to ask the right questions. (Female, aged 35–44 years)

Comments such as these indicate the additional communicative work described by many respondents as necessary to obtain sufficient information from clinical staff. Conversely in the positive comments (143 respondents, 3.1% of free-text respondents), we observe statements indicating satisfaction when information provision and access to specialist staff was perceived to be adequate.

The doctors/surgeons at [hospital removed] were excellent and caring, explaining all that was happen[ing] or about to happen. (Male, aged 65–74 years)

Having access to sufficient information in a timely manner during treatment represents a significant area of concern for many free-text respondents, can be seen as limiting the extra communicative work that some patients perceived in needing to ‘tease information’ from staff, potentially lessening the ‘burden of treatment’.^18^

##### Perceptions of staff manner in interactions with patients

Two hundred and twenty-seven respondents (4.9% of free-text respondents) gave a negative comment about the manner in which they felt that they had been dealt with by healthcare staff. For some, this related to presentation of their diagnosis (54 respondents).

The original time I was told I had terminal cancer and nothing could be done for me was handled very badly. There was no support at all and the doctor was in and out of the room in about 6 minutes. It was as if my life counted for nothing, as if I was being thrown away. (Male, aged 55–64 years)

I took great exception to the manner in which I was told, no privacy, no family member present, and people each side of me could hear. (Female, aged 75–84 years)

Symptoms were observed relating to inadvertent disclosure of cancer diagnosis (of which participants had previously been unaware), as well as not having family or loved ones present when told. Other respondents perceived negative attitudes among consultants and specialists (22 respondents), hospital doctors (35 respondents) or nursing staff (47 respondents).

As a former employee of NHS, I have the greatest respect for the ward staff who work exceedingly hard, but the attitude of some of the medics and other disciplines need to be visited. Sometimes I felt I was treated like a piece of meat or idiot as medics discussed me with colleagues, without ever talking to me directly. (Female, aged 55–64 years)

Some good nurses—but in equal amount, some very lazy, gossiping and bad tempered nurses too. (Female, aged 55–64 years)

However, greater numbers of respondents (n=544) praised the manner of staff during their treatment journey, including *nursing staff* (202 respondents); *hospital doctors* (136 respondents); *consultants and specialist medics* (101 respondents); and *surgical staff* (47 respondents).

The consultant and registrar are most informative and to the point. They always discuss…the way forward with my treatment. I have every confidence in them. (Male, aged 65–74 years)

The nurses that administer the area and in my case carried out tests were very caring and efficient but very obviously overloaded with work. (Male, aged 65–74 years)

Respondent comments highlighted personal qualities (eg, kindness, empathy, sympathy, respect, compassion) in interactions with staff as positive experiences of their treatment, as well as the professionalism of staff involved in their care (eg, that staff were helpful, efficient, competent, etc), despite challenging workloads.

#### Communication between staff and/or institutions

Two hundred and fifty-two respondents commented on interagency/intra-agency communication between staff and/or institutions (eg, information sharing between specialists and GPs where the latter were not identified as the source of the problem, sharing of notes and/or test results between hospitals/sites, etc). Of these, 208 described negative experiences, while 44 gave positive responses (a ratio of 3.8:1). Negative respondents gave general comments relating to this area of communication.

At times, a lack of clear communication between different departments/clinics made the situation more and more difficult….(Female, aged 55–64 years)

Given that this theme references examples of communication in which patients were not involved directly, the generality of the comments is perhaps unsurprising. Nonetheless, they indicate a sizable number of respondents that perceived poor communication between staff and/or institutions involved in their care. Forty-four respondents reported positive experiences, highlighting the beneficial impact that this had on their care.

I appreciate the communication between hospital, GP, out of hours etc. It means I don’t have to repeat myself so often. It also means I have instant treatment. (Female, aged 55–64 years)

Positive respondents often associated perceptions of good communication between staff and/or institutions with speediness of treatment.

### Waiting during the treatment and post-treatment phase

In total, 923 free-text respondents (19.8%) provided a comment about waiting times during the treatment and/or post-treatment phases, either the interval before consultations/treatment (738 respondents, 15.8%) or the time spent waiting in hospitals on the day of appointments (n=194, 4.2%; see online supplementary appendix table S2).

#### 

##### Waiting for appointments

In the closed questions (question 2), 78% of respondents (n=5520) reported they had been seen ‘as soon as necessary’ by an oncologist, with 12% (n=839) feeling that they ‘should have been seen a bit sooner’ and 10% (n=685) indicating that they ‘should have been seen a lot sooner’.^12^ In the free-text portion of the survey, 397 respondents (8.5% of free-text respondents) gave negative comments, while 342 (7.3% of free-text respondents) provided a positive response. The majority of responses in this subtheme did not map to a specific area of treatment or care, but instead referred to consultations or ‘treatment’-related appointments in general terms.

The wait to start treatment is too long. I was initially told I should start treatment by August. I have an appointment for [date removed]. The long delay is disappointing. I was diagnosed in February. (Male, aged 65–74 years)

Positive comments often appeared in the context of broader comments relating to the entire journey.

I went to my GP on the Thursday and I was seen by the following week. The consultant in the hospital which I had biopsies taken and told that same day I had cancer, and it was dealt with very quickly and I was very happy with the care I was given and how quickly it was treated. (Female, aged 55–64 years)

Comments praising the swiftness of appointments during treatment were often attended by expressions of confidence in and satisfaction with the overall package of care given to respondents.

#### Waiting on the day

One hundred and ninety-four respondents (4.1%) provided a comment about waiting times on the day of their appointments during the treatment phase, of which 163 responses (3.5%) were negative and 31 were positive (0.7%). Once again, the majority of these responses were of a general nature, referring only to events such as ‘treatment(s)’ or ‘appointment(s)’. The vast majority of negative comments concerned (unspecified) clinic appointments, and most commonly referred to delays of around 1.5–2 hours beyond the appointed time.

Sometimes as an outpatient with an appointment, the wait is too long! Eg 1 1/2 to 2 hours, even when you arrive well before time. (Female, aged 18–24 years)

It is also important to note that many respondents providing such comments added qualifications indicating their perception of services being under pressure, as a reason for these delays (eg, ‘nurses (were) literally running from one patient to the next’).

A smaller subset of respondents (n=31) reported good or acceptable waiting times on the day of their appointments.

Appointments have been kept on time and in my view within reasonable waiting time. (Male, aged 65–74 years)

While for some waiting times on the day of appointments were not an issue, a greater number of respondents reported difficulties in this area. For some, protracted waiting times were a source of additional problems and discomfort relating to their condition (eg, bladder problems), social and employment commitments and car park charges.

### Staffing and resource levels

Concerns about staffing and resources were expressed by a significant number of free-text respondents (n=671, 14.4%), of which 568 responses (12.2%) were negative, and 107 (2.3%) were positive (see online supplementary appendix table S2). These responses cut across a number of areas of the cancer journey, the largest of which was *availability of aftercare* (312 respondents).

#### Availability of aftercare

This subtheme was comprised of 217 negative and 98 positive responses (ratio 2.21:1). Negative comments identified a lack of general aftercare provision following the completion of treatment, whether chemotherapy, radiotherapy, surgery or other treatment programmes, and this was also true for respondents giving otherwise positive responses.

When discharged from completing radiotherapy I felt quite alone as there had been so much support before. (Female, aged 45–54 years)

The generality of negative comments appears indicative of a profound gap in services after treatment has finished. Support from specialist medical and nursing staff, as well as emotional, social and psychological support while recovering from cancer treatment, were unmet needs reported by many respondents. Concerns included fear of recurrence linked to a lack of clear plans for determining success of treatment, or for long-term monitoring. Several respondents described actual recurrence of cancer, and reported that its discovery was delayed due to failure to conduct what they considered to be appropriate follow-up investigations.

Conversely, comments from the smaller group who provided positive responses reflected experiences of security from regular monitoring, following completion of treatment.

I am being monitored regularly and feel looked after. The specialists are very professional and I felt confident in their care. (Female, aged 55–64 years)

Aftercare was one of the few areas of treatment where negative responses greatly outnumbered positives, and in some cases the former accompanied otherwise positive responses praising many or all other aspects of their cancer journey.

#### General comments about staffing levels (nursing and medical staff)

Two hundred and sixty-seven free-text respondents (5.7%) gave comments relating to staffing levels in hospital settings, of which all but one were negative. While approximately half of respondents (n=141) in this subtheme gave comments of a general nature (eg, referring to ‘staff’ but not specifying a particular specialty), 131 respondents referred to inadequate provision of nursing staff (in general terms), while 17 made (similarly general) comments about hospital doctors.

The nursing staff on the wards work very hard but are very overworked. Staffing levels need to be improved. (Female, aged 55–64 years)

These comments mirror responses to the closed section of the WCPES, in which 29% (n=1229) of respondents indicated that ‘there were sometimes enough (nurses) on duty’; 11% (n=478) indicated that there were rarely or never enough on duty; while 60% (n=2580) agreed that ‘there were always or nearly always enough on duty’.^12^

#### Availability and quality of staff on hospital wards at evening and weekends

Sixty-two respondents provided negative comments regarding out of hours and weekend care with respect to the quality and availability of staff, while eight provided positive responses (ratio 7.5:1). All positive comments were of a general nature (eg, ‘good care at the weekend’). Negative comments presented concerns about staffing levels at weekends and during the night in hospital wards, as well as examples of poor care (again, particularly during the night). Noise levels during the night, and difficulties obtaining out of hours advice and/or treatment for problems arising during treatment were also significant concerns. Some of these comments were general, reflecting concerns around quality of care.

Night time on the ward was awful due to it being short staffed. (Female, aged 65–74 years)

The night staff could have been more respectful it was difficult to sleep because of noise from them, and my bell wasn’t answered. (Female, aged 65–74 years)

These comments were not matched by a significant number of positive comments regarding out of hours/weekend care. While the responses can only reflect the experiences of 60 respondents, the specificity of some of the comments (eg, noise on hospital wards) suggests that there may be specific areas of concern.

#### Availability of specialist nursing staff

Almost all negative comments (n=63, 1.35%) related to availability of specialist nursing staff to answer questions and provide information about treatment.

I felt I needed specialist nurse support (phone or personal contact) following my 3 operations, especially I experienced difficulty with chemotherapy. Needed emotional support, although I did not contact anyone. I live alone and did ask if there was any advice on home support, no action. (Female, aged 55–64 years)

Many respondents perceived specialist nursing staff to be highly pressurised, and linked this perception to lack of availability. However, the majority of comments relating to communication with specialist nursing staff, both in hospital settings (eg, during chemotherapy or radiotherapy treatment) and away from hospitals (eg, district nurse/keyworker visits, access to CNS) were positive (n=106). Indeed, there was a high ratio of positive over negative (1:0.33) comments relating to specialist nurses among free-text respondents.

I have found my nurse specialist to be very helpful and always has time to listen to my concerns. She will always do her best to answer my questions. She always returns calls. (Female, aged 69–74 years)

Comments reflect experiences of high standards in information provision and the manner in which the information was provided by specialist nursing staff, often in spite of significant pressures on their time and resources as perceived by patients.

### Speed and quality of diagnostic care

Four hundred and eleven free-text respondents (8.8%) gave comments relating to the speed and quality of care during the diagnostic phase of the cancer journey, with further comments relating to the *GP role in diagnosis* (n=211) and *investigations and diagnostic procedures* (n=193; see online supplementary appendix table S2).

#### GP role in diagnosis

GPs were the only specialist staff category in which negative free-text responses outnumbered positives (ratio 1.53:1). Among closed, tick-box questions, 78% (n=5520) of respondents reported they had been seen by a hospital doctor as soon as necessary following referral, 12% (n=839) felt that they ‘should have been seen a bit sooner’, and 10% (n=685) ‘a lot sooner’.^19^ Among free-text comments, n=80 (%) respondents described delays in referral by their GP for further investigation of their symptoms, 16 of whom for what they considered an unwillingness to refer.

I had to bypass my GP to get an endoscope test, after numerous requests explaining how ill I felt. The endoscopy dept. discovered the cancer. (Male, aged 75–84 years)

Of particular concern was a subset of respondents (n=35) who described inaccurate diagnosis of their cancer prior to correct diagnosis. This was seen to delay treatment often by months, and in some cases a year or more.

The GP got my condition completely wrong. He had it fixed in his mind that I had haemorrhoids. Finally my daughter took me to A&E, where they discovered an obstruction. The following day I had an endoscopy, which revealed a tumour. That day I had the colostomy. (Male, aged 75–84 years)

In contrast, positive comments (n=52) on GP care tended to be more general, but almost all referred to the speed with which presenting symptoms had been investigated, including referral for further investigations.

The speed at which my GP referred me to a specialist was phenomenal! It gave me a feeling of confidence in the NHS service at a time when I was very frightened. (Male, aged 55–64 years)

Positive responses were often allied with more general comments expressing feelings of satisfaction and reassurance in terms of the overall care and treatment received during the cancer journey.

#### Investigations and diagnostic services

One hundred and seventy-three (3.70%) respondents gave negative comments regarding delays relating to initial diagnostic procedures. Of these, 94 were general or miscellaneous comments regarding delays and/or access to diagnostic services in the initial stages of the cancer journey.

A simple colonoscopy at the early stages would save a lot of pain and suffering and a much cheaper option. (Male, age unavailable)

Thirty-six negative responses included reference to perceived inaccurate or mistaken diagnosis.

I believe the cancer was missed in earlier mammograms. (Female, aged 55–64 years)

While delays and accuracy in diagnostic services were of concern to some, 57 other respondents praised the speed of investigative services.

The speed with which my diagnostic test, scans and surgery were organised. All the doctors exuded a sense of urgency which I found reassuring. (Male, aged 75–84 years)

Delays (whether attributed to waiting times or inaccuracies in diagnosis) were linked to concerns around cancer progression, implications for treatment response, risk of poorer outcomes and additional suffering. Conversely, swiftness of diagnosis was associated with expressions of satisfaction and confidence in the process.

## Discussion

Analysis of free-text comments within the WCPES complements the formal closed questions by allowing patients to indicate the issues most important to them and provides important insights of the experience of patients. The high response rate to the free-text question (64% of those who returned questionnaires) indicates that patients actively engage with the opportunity to provide comments relating to their experiences. They also reflect the findings of the closed questions, that most respondents had a positive overall experience of cancer care. In terms of potential improvement, the themes indicate the impact that uncertainty can have on patient experiences, particularly around perceptions of delays in diagnosis and treatment, or of poor communication during treatment.

For patients who suspect they might have cancer, delay also causes additional psychological distress, which has been shown to correlate positively with the length of that delay.^20^ Previous research has highlighted the presence of free-text comments relating to delays in referral within the CPES England (for London trusts).^14^ Elsewhere it has been indicated that patients are often not satisfied with the time it took for the GP to identify their problem and for a diagnosis to be reached.^21^ Delays for investigations and referral can be caused through ‘misdiagnosis’ with GPs either treating patients symptomatically or relating symptoms to a health problem other than cancer, while for some cancers this could be linked to inadequate patient examination, use of inappropriate tests or failing to follow-up negative or inconclusive test results.^22^ A recent international survey-based study of primary care physicians (PCPs) demonstrated a correlation suggesting a relationship between PCP willingness to act on presentation of symptoms, and cancer survival.^23^ Percentages of PCP respondents in Wales^vi^ that indicated willingness to act on clinical scenarios given in the survey were the lowest for all but two of these scenarios (in which they were second lowest).^23^ These percentages were correlated with survival rates (both 1-year and conditional 5-year survival) that were either lowest or second/joint-second lowest for all of the cancer types.^23^ These findings support patient concerns about a lack of willingness to refer for further investigations at the GP level, which may be indicative of systemic problems at the GP level requiring further investigation. One factor may be communication between and access to support from secondary care, as the authors also reported that PCPs in the UK were the only groups in their study in which most PCPs did not report ready access to secondary care advice about investigation or referral for suspected cancer.^23^

Uncertainty can be understood as a common feature of experiences of patients with cancer, and one that can likely be reduced but not eradicated completely.^24^ ^25^ Our findings indicate consequences of uncertainties for patient experiences in treatment and post-treatment, and areas to which attention may be paid in reducing them. Patients in this study often communicated perceptions of mitigating factors in the issues that they experienced, for example, in highlighting the dedication of staff in circumstances of perceived understaffing. Such comments indicate that where delays and/or uncertainties relating to treatment were present, perceptions of being informed and having a point of contact to ask questions were linked to a greater tolerance for difficulties faced. Recent evidence suggests that patients want more information concerning effects of treatment, and also that patients with cancer continue to receive what they perceive as suboptimal levels of information and preparation.^14^ ^26^ ^27^ A wider range of unmet needs have been identified for those post-treatment or in survivorship relating to emotional and social support, quality of life, long-term functioning and finance.^28–30^ A lack of clarity regarding the process of care has also been identified as an issue for survivors post-treatment, in part associated with less contact with services.^31^ Such support and guidance have been indicated as important factors in patients' satisfaction with their care,^21^ but this requires sufficient and accessible specialist staff. Inadequate staffing levels were perceived as a problem in this study (echoing observations from Wiseman *et al *'s^14^ analysis of CPES England free-text data from London trusts). In the present study, this was particularly true of accessibility of specialist nurses, and recent evidence shows that care coordination and emotional support and support for the control of side effects are better in trusts/hospitals with more specialist nurses.^32^ It is probable that inadequate levels of staffing will also contribute to other problems experienced by patients, such as instances of uncoordinated care, lack of individualised care and waiting for treatment and pain control.^19^

In the post-treatment phase, previous research has indicated that patients can often feel ‘cut adrift’ by the health system after the period of hospital treatment and are left feeling vulnerable and isolated,^33^ a finding echoed by many patients in this study. Evidence indicates that ∼30–50% of cancer survivors have unmet needs, mainly for psychological support and coping with fear of recurrence.^34–36^ While unmet needs reduce for some patients in the months following treatment, one study found that for 60% of these patients the situation did not improve over a 6-month period.^33^ Finally, patients' comments within the WCPES often did not describe specific issues related to aftercare, other than to describe its lack, which reinforces findings from previous studies.^11^ ^14^

Our analysis of the free-text data has been used by Macmillan Cancer Support to gain further insight into the extent and quality of person-centred care in Wales, and to support the organisation's key policy calls for provision of a cancer nurse specialist as the key worker for every patient diagnosed with cancer, as well as a holistic needs assessment and written care plan. It has also formed part of evidence submissions from Macmillan in response to Welsh Government consultations, and the National Assembly's Health and Social Care Committee inquiry, focusing on understanding progress in implementing Welsh Government's Cancer Delivery Plan.

## Study limitations

Data were volunteered by individuals and not reported against a predetermined structured list of topics, and therefore are not necessarily representative. Recall and response bias may also be present. The detail provided in the comments is constrained by the brevity of the response format (ie, a hand-written box) and so may not be as empirically rich as other forms of qualitative data (eg, semistructured interview data or longitudinal diaries). Positive comments tended to be of a more general quality and scope than negative comments, and that a far greater proportion of positive responses were not identified with a specific area (3% of negative respondents vs 22% of positive respondents). Therefore, in more specific categories/themes, numbers of negative respondents may be close to or outnumber positive ones, despite positive responses outnumbering negatives overall. Counts relating to comments refer to numbers of respondents providing comments in specific categories/themes, and as such negative and positive comments in a given area may not equal the total amount of respondents (ie, because individual respondents may have provided both negative and positive comments). Counts do not account for the strength of comments or their seriousness (eg, a negative comment concerning quality of meals counts towards a total in the same way as a more serious symptom relating to poor care or treatment).

## Future research

Manual coding of free-text affords the most thorough means to analyse these data thematically; however, working with a large corpus is a labour-intensive process, and larger projects may require additional methods for manipulation and sorting of free text, in order to produce thematic analyses at the level of detail in the present study. Our previous work with colleagues on survey data from patients with colorectal cancer has used text mining techniques to automate sorting of responses into broad categories for manual coding.^37^ In addition, the PRESENT (Patient Reported Experience Survey Engineering of Natural Text) project currently underway will explore and develop methods for working with these data using text engineering.^38^

In their analysis of England CPES free-text data from patients with cancer within London NHS trusts, Wiseman *et al*^14^ noted that a number of patients described care outside of their assigned trusts, and/or sought to identify closed-question responses with areas outside of London. Both types of response were observed in the present study, and therefore future research might seek to examine associations between specific treatment sites and responses. Such work would be of benefit in assessing and developing the ability of surveys such as CPES to reflect the complexities of cancer care pathways.^14^

## Conclusion

This study has illustrated the value of free-text analysis for exploring patient experiences of cancer care, and for complementing and extending findings from closed questions. As the first systematic analysis of free-text data from a national sample of experiences of patients with cancer, it has presented specific areas of concern for patients with cancer, as well as areas of good practice, and revealed themes present across the cancer journey. The volume of comments within specific themes, as well as ratios of negative-to-positive comments, indicate areas of potential concern. Our work on the WCPES has also highlighted an area of potential significance with regard to the reliability of survey data at greater levels of specificity (ie, the site level). These findings have been discussed in the context of existing issues in cancer care, and in doing so have presented areas of specific attention for policymakers and further research.

10.1136/bmjopen-2016-011830.supp2 Supplementary data

## Footnotes

i Data definitions identifying patients qualifying for inclusion were based on the International Classification of Diseases (ICD)10 codes of C00-C99, and D05 were used. Patients with an ICD10 code of C44 (other malignant neoplasms of the skin), and C84 (some haematology codes) were excluded from the sample by agreement. There are only very small numbers of such patients with these codes.^16^

ii n=7190.

iii n=162.

iv Respondent/response counts in this article refer to incidence of individual respondents within a given code or theme (ie, a respondent would only be counted once towards the total for ‘surgery positive’ even if the comment referred to two different incidents of surgical care).

v A full breakdown of the stage 2 analysis is included as an online supplementary file.

vi Approximately n=217 participants (11.7% crude response rate, 1861 invited).^1^,^9^
