# Supplementary material for: Exploring experiences of cancer care in Wales: a thematic analysis of free-text responses to the 2013 Wales Cancer Patient Experience Survey (WCPES)
Source: BMJ Open. 2016 Sep 1;6(9):e011830. doi: 10.1136/bmjopen-2016-011830 (PMC5020838; doi:10.1136/bmjopen-2016-011830)
Supplement: Supplementary tables [file bmjopen-2016-011830supp_tables.pdf]

Appendix

Table 1 – Results of Stage 1 analysis - no. of respondents providing positive and negative comments relating to categories of cancer patient experience.

Category	*Negative respondents*	*Negative respondents as % of total category respondents*	*Positive respondents*	*Positive respondents as % of total category respondents*	*Total respondents*[1]	*Ratio (negative-to-positive comments, n : 1)*
Waiting for appointments	397	54%	342	46%	738	1.16
Communication between patients and staff (NOS[2])	287	52%	287	52%	554	1
Surgery	181	33%	393	73%	541	0.46
General nursing	127	25%	402	78%	517	0.32
Hospital doctors	73	15%	411	86%	476	0.18
Investigations and diagnostic services	288	61%	198	42%	475	1.45
Consultants and specialists	72	15%	408	88%	465	0.18
Specialist nursing	108	25%	329	76%	433	0.33
GP	246	61%	161	40%	401	1.53
Chemotherapy	85	28%	233	77%	303	0.36
Aftercare (NOS)	199	69%	97	33%	290	2.05
Radiotherapy	67	27%	191	76%	251	0.35
Hospital environments	182	76%	53	22%	240	3.43
Communication between staff and/or institutions (NOS)	165	80%	44	21%	206	3.75
Waiting to be seen on the day	163	84%	31	16%	194	5.25
Travel-related issues during the cancer journey	122	76%	45	28%	161	2.71
Food and catering	128	84%	26	17%	153	4.94
Emotional, social, psychological support	94	71%	43	33%	132	2.19
Concerns about staffing levels	131	100%	-	-	131	-

[1] As individual patients may have provided both a negative and a positive comment in a given area – the total number of respondents for a given category may be less than the sum of positive and negative respondents (i.e. each respondent would only be counted once for the area as a whole). Combined positive and negative percentage therefore may not equal 100%.
[2] Not otherwise specified – comments in these categories refer to generic aspects of experience (e.g. communication), which were not associated with a specific area of treatment or care (e.g. surgery).

Oncology	31	26%	90	77%	117	0.34
Pain management	73	89%	10	12%	82	7.3
Out of hours and weekend care	60	88%	8	12%	68	7.50
Accident & Emergency	33	80%	8	20%	41	4.13
Urology	7	18%	34	87%	39	0.21
Financial concerns	35	97%	3	8%	36	11.67
Physiotherapy	12	50%	12	50%	24	1
Total	***2313***	50%	***3818***	82%	***4672***	***0.60:1***

Table 2 - Results of Stage 3 analysis - number and percentages of respondents by theme/sub-theme

Theme	Sub-themes	Negative respondents (n)	Negative respondents (% of free-text respondents)	Positive respondents (n)	Positive respondents (% of free-text respondents)	Total respondents (n)	Total respondents (% of free-text respondents)	Total respondents (% of survey respondents)	Ratio (negative-to-positive, n : 1)
***Communication*[3]**	Communication between patients and staff[4]	687	14.70%	809	17.32%	1472	31.51%	20.02%	0.85
	Communication between staff and/or institutions	208	4.45%	44	0.94%	252	5.39%	3.43%	4.73
	Communication totals	*854*	*18.28%*	*846*	*18.11%*	*1673*	*35.81%*	*22.76%*	*1.01*
Waiting during the treatment and post-treatment phases	Waiting for appointments	397	8.50%	342	7.32%	738	15.80%	10.04%	1.16
	Waiting on the day	163	3.49%	31	0.66%	194	4.15%	2.64%	5.26
	Waiting totals	*522*	*11.17%*	*372*	7.96%	*923*	*19.76%*	*12.55%*	*1.40*
Staffing and resource levels	Availability of aftercare[5]	217	4.64%	98	2.10%	312	6.68%	4.24%	2.21
	General comments about staffing levels (nursing and medical staff NOS)	266	5.69%	1	0.02%	267	5.71%	3.63%	266.00
	Availability and quality of staff on hospital wards at evening and weekends	62	1.33%	8	0.17%	70	1.50%	0.95%	7.75
	Availability of specialist nursing staff	63	1.35%	.	.	63	1.35%	0.86%	.
	Staffing and resource levels totals	*568*	*12.16%*	*107*	*2.29%*	*671*	*14.36%*	*0.86%*	*5.31*
Speed and quality of diagnostic care	GP role in diagnosis	159	3.40%	52	1.11%	211	4.52%	2.87%	3.06
	Investigations and diagnostic procedures[6]	173	3.70%	57	1.22%	193	4.13%	2.63%	3.04
	Speed and quality of diagnostic care totals	314	*6.72%*	97	2.08%	411	*8.80%*	5.59%	3.24
Totals		***1948***	***41.70%***	***1276***	***27.31%***	***3172***	***67.89%***	***43.14%***	***1.53***

[3] Sub-themes in this theme together all sub-categories of communication between patients and staff, whether NOS or belonging to a specific area of care or treatment (e.g. surgery).

[5] This sub theme collects all NOS and specific sub-categories relating to availability of aftercare.

[6] This sub-theme contains only comments relating to quality and speed of investigations and diagnostic services – other comments are excluded (see table 2).

Table 3 - Respondent demographics.[7]

Demographic category	*Demographic sub-category*	*Number of free-text respondents / as % of total free-text respondents (n = 4672)*	*Number of survey respondents / % of total survey respondents (n = 7352)*
**Tumour Group**	Breast	1168 / 25%	1717 / 23%
	Colorectal / Lower Gastrointestinal	722 / 15%	1112 / 15%
	Prostate	587 / 13%	954 / 13%
	Urological	455 / 10%	787 / 11%
	Haematological	405 / 9%	633 / 9%
	Gynaecological	340 / 7%	504 / 7%
	Lung	238 / 5%	427 / 6%
	Head and Neck	212 / 5%	332 / 5%
	Upper Gastrointestinal	210 / 4%	354 / 5%
	Other	161 / 3%	252 / 3%
	Skin	99 / 2%	163 / 2%
	Sarcoma	46 / 1%	64 / 1%
	Brain/Central Nervous System	29 / 1%	53 / 1%
**Health Board**	Velindre NHS Trust	1283 / 27%	2053 / 28%
	Betsi Cadwaladr University Health Board	1097 / 23%	1720 / 23%
	Abertawe Bro Morgannwg University Health Board	962 / 21%	1539 / 21%
	Hywel Dda University Health Board	699 / 15%	1069 / 15%
	Cardiff And Vale University Health Board	256 / 5%	384 / 5%
	Cwm Taf University Health Board	189 / 4%	307 / 4%
	Aneurin Bevan University Health Board	186 / 4%	280 / 4%
**Sex**	Male	2065 / 43%	3397 / 46%
	Female	2522 / 53%	3785 / 51%
	Data not available	85 / 2%	170 / 2%
**Approximate age range (years)**	95-99	9 / 0.2%	12 / 0.2%
	85-94	188 / 4%	332 / 5%
	75-84	1022 / 22%	1656 / 23%
	65-74	1509 / 32%	2315 / 31%
	55-64	920 / 13%	1386 / 19%
	45-54	428 / 11%	616 / 8%
	35-44	119 / 8%	178 / 2%
	25-34	41 / 6%	66 / 1%
	<=24	7 / 2%	15 / 0.2%
	Data unavailable	429 / 9%	776 / 11%
**Sexual Orientation**	Heterosexual / straight (opposite sex)	4275 / 92%	6595 / 90%
	Data not available	231 / 5%	459 / 6%
	Prefer not to answer	117 / 3%	222 / 3%
	Gay or Lesbian (same sex)	21 / 0.4%	27 / 0.4%
	Bisexual (both sexes)	14 / 0.3%	19 / 0.3%

[7] Percentages for each demographic category may not add up to 100% due to rounding area.

	Other	14 / 0.3%	30 / 0.4%
**Main employment status**	Retired	2892 / 62%	4608 / 63%
	Full time employment	715 / 15%	1056 / 14%
	Part time employment	334 / 7%	497 / 7%
	Unemployed – unable to work for health reasons	300 / 6%	494 / 7%
	Data not available	161 / 3%	266 / 4%
	Homemaker	127 / 3%	206 / 3%
	Other	108 / 2%	174 / 2%
	Unemployed – and seeking work	23 / 0.5%	36 / 0.5%
	Student (in education)	12 / 0.3%	15 / 0.2%
**Longstanding conditions**	Do not have long term condition	2631 / 56%	4082 / 56%
	Long-standing physical conditions	865 / 19%	1291 / 18%
	Long-standing illnesses, such as HIV diabetes, chronic heart disease, or epilepsy.	660 / 14%	1023 / 14%
	Deafness or severe hearing impairment	504 / 11%	852 / 12%
	Mental health conditions	117 / 3%	187 / 3%
	Blindness or partially sighted	106 / 2%	67 / 1%
	Learning disabilities	17 / 0.4%	31 / 0.4%
**Ethnicity**	White (British)	4467 / 96%	6991 / 95%
	White (Irish)	27 / 0.58%	44 / 1%
	White (other)	64 / 1%	102 / 1%
	Mixed (White and Black Carribean)	5 / 0.11%	7 / 0.1%
	Mixed (White and Black African)	1 / 0.02%	4 / 0.1%
	Mixed (White and Asian)	3 / 0.06%	4 / 0.1%
	Mixed (other)	2 / 0.04%	2 / 0.03%
	Indian	6 / 0.13%	7 / 0.1%
	Pakistani	3 / 0.06%	5 / 0.1%
	Bangladeshi	0 / 0.00%	1 / 0.01%
	Asian (other)	4 / 0.09%	6 / 0.1%
	Carribean	2 / 0.04%	3 / 0.04%
	African	4 / 0.09%	5 / 0.1%
	Black (other)	0 / 0.00%	0 / 0.00%
	Chinese	1 / 0.02%	5 / 0.1%
	Any other ethnic group	3 / 0.06%	4 / 0.1%
	Data unavailable	80 / 2%	162 / 2%
