# Supplementary material for: Exploring experiences of cancer care in Wales: a thematic analysis of free-text responses to the 2013 Wales Cancer Patient Experience Survey (WCPES)
Source: BMJ Open. 2016 Sep 1;6(9):e011830. doi: 10.1136/bmjopen-2016-011830 (PMC5020838; doi:10.1136/bmjopen-2016-011830)
Supplement: Supplementary data [file bmjopen-2016-011830supp.pdf]

Category/sub-category	Respondents
Admin	117
AdminImprove	74
AdminPositive	43
Carers-Dependents-Family	27
CarDepFamImprove	24
CarDepFamPositive	2
Comorbidities	35
ComorbiditiesImprove	31
ComorbiditiesPositive	5
Facility closures	22
Finances	37
FinancesImprove	35
FinancesPositive	3
Travel	170
After care	312
AftercareImprove	217
AftercareE-S-MH-Improve	19
AftercareImproveNOS	170
InvestFollowImprove	28
AftercarePositive	98
AftercarePositiveNOS	83
InvestFollowPositive	17
BreastCare	157
Dressing-wound Care	32
Dress-woundCareImprove	29
Dress-woundCarePositive	3
EarlyDischarge	1
Emotional-Social-MH support	143
Emotional-Social-MHImprove	100
EmSocMHAfterCareImprove	19
Emotional-Social-MHPositive	43
Environment	265
CleaningStaff	22
EnvironmentImprove	195
EnvBedLevelsImprove	60
EnvHospCleanImprove	22
EnvHospToiletImprove	16
EnvHospPrivacyImprove	22
EnvironmentPositive	53
BedLevelsPositive	1
EnvHospCleanPositive	18
Food-Catering	159
FoodCatImprove	132
FoodCatPositive	27
Hospital Infections	26
Nursing	1193
NursingImprove	421
NursAreas	102
NursAvailImprove(NOS)	32
NursComImprove	71
NursComImproveNOS	49
NursInfoImproveNOS	4
NursMannerImproveNOS	47
NursInfoImprove	7
NursMannerImprove	65
NursDisciplinesImprove	108
NursBreastImprove	19
NursBreastAvailImprove	8

NursCNSImprove	11
NursCNSAvailImprove	8
NursDistrictImprove	29
NursDistAvailImprove	11
NursKeyImprove	20
NursKeyAvailImprove	18
NursMacMilImprove	18
NursMacMilAvailImprove	9
NursSpecialImprove(NOS)	16
NursSpecialAvailImprove	13
NursImproveNOS	132
NursCareImproveNOS	72
NursOutOfHoursImprove	24
NursLevelsImprove	131
NursingPositive	811
NursComPositive	249
NursComPositiveNOS	2
NursInfoPositive	66
NursInfoPositiveNOS	38
NursMannerPositive	202
NursMannerPositiveNOS	153
NursDisciplinesPositive	329
NursBreastPositive	51
NursBreastManner-InfoPositive	18
NursChemoPositive	69
NursChemoInfoPositive	8
NursChemoMannerPositive	25
NursCNSPositive	45
NursCNSManner-InfoPositive	18
NursDistPositive	48
NursDistMannerPositive	10
NursKeyPositive	18
NursMacMilPositive	45
NursMacMilManner-InfoPositive	14
NursSpecialPositive(NOS)	70
NursSpecialInfoPositive(NOS)	6
NursSpecialMannerPositive(NOS)	19
NursPositiveNOS	410
Out of hours-Weekend(NOS)	71
OutofHours-WeekendImprove(NOS)	62
OutofHours-WeekendPositive(NOS)	8
Palliative Care	17
PalliativeCareImprove	4
PalliativeCarePositive	13
Staffing-Resource Levels	144
StaffingLevels(NOS)	142
StaffingLevelsImprove(NOS)	141
StaffingLevelsPositive(NOS)	1
Stoma	8
A&E	41
A&EImprove	33
A&EPositive	8
Anaesthesia	22
AnaesImprove	6
AnaesPositive	16
Chemotherapy	324
ChemoImprove	89
ChemoComImprove	28
ChemoInfoImprove	27

ChemoImproveNOS	61
ChemoPositive	236
ChemoComPositive	61
ChemoInfoPositive	15
ChemoMannerPositive	49
ChemoPositiveNOS	177
Choice-Treatment Options	64
Choice-TreatOpImprove	49
ChoiceTreatInfoDisImprove	27
Choice-TreatOpPositive	14
Clinical Trials	34
Communication	1377
CommunicationInterIntra-agency	252
CommunicationInterIntra-agencyImprove	208
ComInterIntraAgencyImproveNOS	171
NoteLossImproveNOS	15
CommunicationInterIntra-agencyPositiveNOS	44
CommunicationPatient-Provider	1171
CommunicationPatient-ProviderImprove	619
ComPatProvImproveNOS	315
InfoNOS	152
InfAfterNOS	8
InfDiagNOS	8
InfTreatNOS	62
MannerNOS	96
LangNOS	14
ManDiagNOS	57
CommunicationPatient-ProviderPositive	569
ComPatProvPosNOS	292
InfoPosNOS	77
InfoPosNOSMaterials	7
InfoPosNOSTelephone	12
ManDiagPosNOS	1
MannerPosNOS	217
MannerPersPosNOS	194
MannerProfPosNOS	74
Consultants-SpecialistsNOS	487
ConsultSpecialImprove	75
ConSpecAppSpeedImprove	11
ConSpecComImprove	47
ConSpecInfoImprove	25
ConSpecMannerImprove	22
ConsultSpecialPositive	415
ConSpecAppSpeedPositive	11
ConSpecComPositive	133
ConSpecAccessPositive	10
ConSpecInfoPositive	45
ConSpecMannerPositive	101
Dermatology	12
ENT	14
ENTImprove	6
ENTPositive	8
GP	422
GPImprove	258
GPCarePDiagImprove	70
GPCondKnowImprove	18
GPServProvImprove	8
GPDiagImprove	159
GPDiagComImprove	13

GPDiagSpeedImprove	39
GPMisdiagImprove	35
GPReferralImprove	80
GPImproveNOS	33
GPPostive	165
GPDiagPositive	52
GPReferralPositive	41
GPPDiagCarePositive	43
GPPositiveNOS	70
Gynaecology	12
GynaeImprove	5
GynaePositive	7
Haematology	27
HaemImprove	2
HaemPositive	25
HospitalDoctorsNOS	499
HospitalDoctorsImprove	79
HospDocComImprove	51
HospDocInfoImprove	26
HospDocLangImprove	4
HospDocMannerImprove	35
HospDocLevelsImprove	17
HospitalDoctorsPositive	422
HospDocComPositive	145
HospDocInfoPositive	11
HospDocMannerPositive	136
IntensiveCare	10
Investigations	502
InvestigationsImprove	306
InvestImproveNOS	104
InvestSpeedImprove	136
InvestInitialImprove	58
InvestMisImprove	37
WaitingResultsImprove	77
DiagWaitNOS	41
FollowResultsImproveNOS	4
TreatResultImproveNOS	9
WaitingResultsPositive	5
InvestigationsPositive	202
InvestPositiveNOS	81
InvestSpeedPositive	57
ScreeningPositive	65
ScreenBowelPos	25
ScreenBreastPos	28
Lymphodema	32
LymphoImprove	19
LymphoPositive	15
Oncology	123
OncologyImprove	32
OncComImprove	20
OncInfoImprove	13
OncMannerImprove	7
OncologyPositive	94
OncComPositive	16
OncAccessPositive	2
OncInfoPositive	7
OncMannerPositive	10
OtherTreatments	50
OtherTreatImprove	35

OtherTreatPositive	15
PainManagement	91
PainManageImprove	82
PainChronicImprove	11
PainDischImprove	8
PainWaitImprove	29
PainManagePositive	10
Pharmacy-Medication	80
PharmacyMedicationImprove	59
PharMedInfoImprove	5
PharmacyMedicationPositive	21
Physiotherapy	35
PhysioImprove	12
PhysioPositive	13
PrivateTreatment-Care	89
PrivAfterCare	2
PrivDiag	59
PrivDiagAvoidDelay	45
PrivTreatCare	45
PrivNHSUnavail-Wait	18
PrivTreatCareImprove	4
PrivTreatCarePositive	23
Radiotherapy	265
RadiotherapyImprove	71
RadComImprove	29
RadInfoImprove	24
RadMannerImprove	6
RadImproveNOS	42
RadiotherapyPositive	195
RadComPositive	74
RadInfoPositive	20
RadMannerPositive	65
RadPositiveNOS	121
Respiratory	19
RespImprove	6
RespPositive	14
Surgery	589
SurgeryImprove	190
SurgCancelDelayImprove	20
SurgComImprove	68
SurgInfoImprove	36
SurgLangImprove	3
SurgMannerImprove	16
SurgeryImproveNOS	18
SurgFollowImprove	17
SurgPostOpImprove	54
PainPostOpImprove	16
SurgPostOpRecImprove	49
SurgPreOpImprove	18
SurgProcedImprove	15
SurgeryPositive	407
SurgAppointSpeedPositive	78
SurgComPositive	63
SurgInfoPositive	19
SurgMannerPositive	47
SurgeryPositiveNOS	190
SurgPostOpPositive	46
SurgPostOpRecPositive	38
SurgPreOpPositive	20

SurgProcedPositive	61
Urology	44
UrologyImprove	7
UrologyPositive	37
Waiting Times	930
